# New Jersey State Dental Society

**Published:** 1890-02

**Authors:** 


### NEW JERSEY STATE DENTAL SOCIETY.—(*Concluded.*)

### *Wednesday, July 19, 1889.—Evening Session.*

THE roll was called, forty-two members present.

Dr. Watkins, chairman of the Executive Committee, reported that that committee had agreed to make an assessment upon members, for the purpose of defraying the expense of publishing the proceedings of the society for the first five years of its existence. Also, that, as Dr. Sudduth has offered to reprint, free of cost, one hundred and fifty copies of the proceedings of the society, the INTERNATIONAL DENTAL JOURNAL be made the official organ of the society for this year.

On motion the report was received.

*Dr. G. Carleton Brown.*—Mr. President, in regard to the assessment ordered at the last meeting and made by the Executive Committee, I would state that it has been found necessary to make this assessment larger than usual,—four dollars. There are five years' proceedings included in this publication, and it brings the literature of the society up to date. The expense has been met by the officers of the society; and we would now ask that the members of the society make this good to them by the prompt payment of their dues and assessments.

Elbert T. Davis, D.D.S., of Bridgeton, was proposed for active membership; endorsed by Drs. Richards and Meeker. Also Dr. A. Westlake, of Elizabeth, endorsed by Drs. Levy and G. Carleton Brown.

The nominations were ordered to take the usual course.

The secretary announced that he had received the credentials of Dr. L. Ashley Faught as a delegate from the Philadelphia County Dental Society, of Pennsylvania.

Dr. Wm. H. Dwinelle, of New York, read a paper on the treatment of teeth during pregnancy. (See page 65.)

### DISCUSSION OF DR. DWINELLE'S PAPER ON "TREATMENT OF THE TEETH DURING PREGNANCY."

*Dr. E. T. Darby.*—Mr. President, there is very much of truth in what Dr. Dwinelle has read to us this evening. We all know that the period of pregnancy is one peculiarly trying upon a woman.

We also know that during the period of pregnancy the nervous system is wrought up to its highest tension. We know that at this period there is often a demand in the system for lime salts. This has been illustrated in various ways, as by the mother craving at such times food that contains the phosphates in abundance. I think my friend, Dr. Kirk, some years ago read a paper upon this subject, stating for illustration several cases in which the women during this period had craved lime to such an extent that they had even eaten mortar and lime from the boxes in the street. I have known instances of pregnant women who ate plaster from the houses, and also ate chalk in large quantities. This seems proof positive that there was in the system a craving for lime. It has been questioned by many scientific men, and by men in our own profession, whether any change takes place in the teeth in consequence of the introduction of lime salts into the system. I have heard Professor Black say that a tooth once soft is always soft; that no change takes place in that tooth by the introduction of lime salts into the system. I differ very materially from Dr. Black upon this point, because I am positive that I have seen changes take place in teeth for the better; teeth that were soft in early life becoming hard and good later in life. I have also seen in my own practice cases where a diet composed largely of phosphates was introduced, where the diet had been of a very different character, containing little phosphates, and the teeth being in bad condition; and the favorable results upon the teeth following that change in diet were truly wonderful. I think it was Ben Jonson who said that the oatmeal of Scotland had made good men in England, as it had made good horses. I think if we could introduce into the system of women during the period of gestation a greater proportion of the phosphates in their food, there would be a great improvement in the teeth of their children. But, unfortunately, these pregnant women do not take to oatmeal and brown bread and such things, but more frequently crave those things which Dr. Dwinelle says are inert in the system, such as lime, which is not assimilated. In the treatment of the teeth of pregnant women I think we sometimes insist upon too radical treatment, trying to perform operations which we ought not to perform under those conditions. My own practice has been, during the period of pregnancy, to fill the teeth with plastics only, as much as possible avoiding large operations and the use of metallic substances. It is not, in acid conditions, perhaps the best thing to use oxyphosphate or oxychloride; gutta-percha is preferable, because the secretions are intensely acid at that time. If we would perform only temporary operations during the period of gestation the results would be more satisfactory.

I have in my practice a patient whose teeth I have had the care of from the time she was five or six years old. She has been married about five years, and has had three children. Previous to her marriage she had very little dentistry to do, her teeth being of unusually good character. I saw her a few weeks ago, and her teeth were in wretched condition. During these three pregnancies she has not had time, or has not been in condition, to give her teeth the attention she did previously, and the ravages of decay have been alarming. It is unquestionably the result of her frequently renewed pregnancy.

It is very difficult to know just what is the best treatment in these conditions. It has been asserted that the system of the woman is drawn upon at this period for an unusual quantity of lime salts to build up the teeth of the fœtus, and that the introduction of phosphates would be beneficial.

*Dr. Littig.*—Is it the phosphate or phosphite that we want?

*Dr. Darby.*—I have been under the impression that it was the phosphate.

*Dr. Littig.*—I think it is the phosphate that the system throws off, and the phosphite is used up by the addition of oxygen. When we have the phosphate, which has an atom of phosphorus and an atom of oxygen, they take on another atom of oxygen and we have the phosphite. The phosphite has three atoms of oxygen and one of phosphorus. The phosphate has five atoms of oxygen. As nature uses the phosphite, oxygen is added until it takes on its full equivalent of oxygen, and then it becomes a phosphate, and nature throws it off. Am I right?

*Dr. Darby.*—I cannot say whether you are chemically correct or not.

*Dr. Littig.*—I think the craving for chalk and lime is simply on account of the acid condition. The acidity is corrected by taking the lime, but it does not really enter into the system. I have an idea that the thing needed is the phosphite, which is in the grain; because, if I wanted to raise a plant I would put phosphate on it. The plant, in its growth, takes up one atom of oxygen and another atom of oxygen until it makes up the phosphate and leaves the phosphite in the grain. I believe it is the phosphite we want and not the phosphate; that the phosphate is inert all the way through. I do not pretend to be a chemist, but that seems to be the natural condition of things.

*Dr. Darby.*—I have in mind a case of a woman whose teeth were going very rapidly during her pregnancy. I said to her physician one day, "This lady's teeth are decaying very rapidly; what is your opinion in regard to the use of hypophosphites?" He said, "I have a very good opinion of them, and, if you agree with me, I will prescribe them for her." He put her upon cod-liver oil and hypophosphites; and he seemed to think she was very much better physically, and I do not know but that her teeth were very much improved by that treatment.

*Dr. Littig.*—I believe that if you could get phosphite and keep it pure it would be beneficial; but as soon as you open any article that contains phosphite, the introduction of oxygen disrupts it and you have the phosphate again; so it is of very little service after it has been opened for any length of time.

*Dr. Kirk.*—Does Dr. Littig understand that the phosphoric compounds to be appropriated by the organism must be necessarily in the form of phosphites and not phosphates?

*Dr. Littig.*—I think it is the phosphites.

*Dr. Kirk.*—I think that is incorrect. In the outer shell of the wheat, I think, you will find the lime salts exist as phosphates as well as phosphites. It is a very interesting point that Dr. Littig has raised, as to whether the phosphates in the system are produced by oxidation of the phosphites taken in as food; but I do not think there is any evidence to disprove that the phosphates, as they exist in the various articles of food when taken into the system, are directly appropriated. It is quite a new thought to me, this question, in regard to the assimilation of phosphates. I have always believed that the phosphates, as they exist in the grain or muscle or bony tissue taken as food, are directly appropriated by the organism they are taken into.

*Dr. Littig.*—I would not undertake to dispute Dr. Kirk on a question of chemistry. But I wish he would look into this subject; perhaps he will find I am a little nearer right than he thinks.

*Dr. Kirk.*—In relation to Dr. Dwinelle's paper, there are a number of points in it that have been intensely interesting to me for many years. One is the question of whether the tooth-structure in the mother deteriorates during pregnancy, or whether the caries that runs riot during the period of gestation is produced by conditions dependent upon the local production of acids. I have always believed, and shall continue to believe as long as I have a tactile sense sufficiently delicate to distinguish between hard and soft dentine, that the tooth-structure during pregnancy softens. At the same time there is, apparently, an acid condition of the secretions, brought about by changes in the systemic condition of the patient during that time. I have in mind the case of a lady that came to me about the time of her marriage, her teeth then being in nearly perfect condition. She has since then given birth to a child almost annually for the past four or five years; and the result is excessive erosion of the surfaces of all the teeth anterior to the molars, on their labial and buccal aspects. It has been almost impossible to prevent this destruction of the tooth substance; and I told her she would have to choose between having babies or teeth, as she could not have both. I want to say that, in my opinion, the habit that many women have of giving birth to a baby once a year is a very bad one, and, from the stand-point of dental prophylaxis alone, ought to be corrected.

*Dr. Dwinelle.*—I hope the doctor will give us a remedy.

*Dr. Kirk.*—If there is any one who absolutely needs a remedy, I will give it to him.

*Dr. W. S. Elliott.*—The subject before us is one of considerable interest. The paper suggests this thought,—that the desire for an antacid lies with the oxide of calcium; from which we could presume that it was oxide of calcium, or common lime, that the patient needed. But I think, as the gentleman from New York has said, that it is more to correct the acid condition that the system demands lime; because we know that common lime is nothing more than oxide of calcium, and is not assimilable as such.

As regards the difference between the bone phosphates and the inert phosphates of the rocks, I do not know what the chemical difference is. I do not believe there is really any difference from this point of consideration,—that all the lime of the earth, as naturalists understand it, has at one time been part of the living animal; that all the lime of the earth has really been in solution in the sea, and has been appropriated by animal life and then deposited in the rocks. Therefore it has been vitalized. Whether the lapse of ages has deprived it of that vitality or not is a question I could not answer; but it is generally understood that all lime has once been vitalized by passing through a vital medium. I believe the element which the system really requires in the condition of pregnancy is that of the hypophosphites. As far as the grain is concerned, there is a difference between the outer hull, or husk, and that layer which lies between the centre and the husk, and which contains more of the phosphites than the outer shell; and it is the phosphites of the kernel which is appropriated by the system, and not the phosphate.

*Dr. Dwinelle.*—In regard to the comparative potency of animal phosphates and phosphates derived from the rocks, we have a splendid illustration in medico-legal history, as derived from a great fact that was brought to light in England many years ago. I can not recall the names to enforce what I am about to say. We will assume that there was once upon a time a certain Jaynes's powder, that was very potent and efficacious in curing the malignant malarious fevers of the extreme East, where England had her armies by hundreds of thousands. Annually there broke out in that region occupied by the English troops this malignant fever, which devastated the army more than battles, unless they had a quantity of Jaynes's powder to combat it and allay its ravages. Jaynes's powder was a potent and effective remedy. Thus matters went on until, in the course of time, many generations perhaps, the Jayneses became immensely rich; with riches came education, intelligence, and refinement; and in course of time there was an appeal put by the country at large to have made known the components of this powder, which was a secret up to that time; there was an appeal to the generosity, intelligence, and fairness of the manufacturers of this powder to make known the secret of its preparation. "Certainly, gentlemen," said they to the committee, "we have for a long period of time derived great advantage, wealth, and power from it, and it is no more than reasonable that we should do as you request; we ought to have done it long ago; we have not the right to keep a process secret which belongs to humanity." So they published the recipe to the world; whereupon there were bids made immediately for supplying Jaynes's powder for the East. Among others Jaynes was one of the bidders, but he was underbid by one Jones, and Jones got the contract. Jaynes came forward and said to Jones, "I congratulate you; all of my apparatus and plant, my factories, my stock, my experts, and my men are at your service." The offer was accepted, on certain terms, and Jones went to work to make the Jaynes powder, seeing great success and immense wealth before him. But his dream was soon ended. The fever broke out as usual, and the Jaynes-Jones powder was administered as before, but it had no effect, it was wholly inert, and the soldiers and others died like sheep with the rot. The result was alarming; the army was rapidly reduced, and the whole mother country was stirred to desperation by the terrible intelligence that came to them from the East. The matter was brought before Parliament, and an investigation was made. Jaynes came forward and rendered every assistance that he could in the premises, as did others. Jones was confounded. The old Jaynes experts were put upon the witness-stand, and questioned closely in reference to any difference between the two recipes, and they declared them to be the same; each contained so much phosphate of lime, and so much of this, that, and the other. They were confounded, and the inquiry arose, What shall we do to be saved? The investigation went on day after day. The old experts were put upon the stand again and again; each time they declared that the materials used by Jones were precisely the same in nature and in quantity as those which had been used by Jaynes. Finally the counsel in the case said, "You say that Jaynes's powder contained so much phosphate of lime?" "Yes, sir." "And you say that Jones's powder contains precisely the same quantity of the same material?" "Yes, sir." "Where did Jaynes get his phosphate of lime?" "Why, from the bones of animals." "Where did Jones get his?" "He got his from the phosphate rocks; but they are precisely the same, chemically." The counsel sprang to his feet and cried, "Eureka! I have found it.

"In the beginning a nebulous sphere, containing all of the elements, millions of miles in diameter, transparent as ether, was floating in space. Suddenly, through the potency of electric force, this immense area was condensed into the circumference of a few thousand miles, and hurled into its prescribed orbit in the form of a ball of fire.

"From the elements, air and water took their appropriate place, upheavals forced elemental rocks above the surface, separating land from sea,—among them the phosphate rocks.

"It took the phosphate rock unnumbered millions of ages—passing through the alembic of nature's laboratory, through the stomach of myriads of animals, through an infinite and ever-advancing vegetation, devoured and digested by animals, through the cold-blooded range, ever evolving still higher and higher, up through the varied types of the warm-blooded mammalia—before its primitive and inert condition had become so far *animalized* and *spiritualized* that it would be acceptable as a pap, a pabulum to be assimilated with the human economy in the form of phosphates derived from the bones of animals.

"Phosphate of lime derived from the phosphate rock has no affinity with our animal economy; it is wholly inert, fatally inert, as proven by the unhappy experience of the Joneses in India, and established by this court of inquiry. The phosphate of lime obtained from the bones of the mammalia, through the mysterious processes of untold ages, readily assimilates with our elemental humanity, and is potent to save, as proven by the record of the honored Jayneses for generations past, all of which has been amply verified by this long and laborious investigation.

"The great lesson of this hour of our solemn victory bids us take heed that we recognize the fact that we live in an age where subtle, potent, and hitherto unknown elements and conditions challenge our recognition."

In the course of time Jones adopted the formula of Jaynes, and his powders were potent ever after.

At the time of the great fatality in India, some of the original Jaynes powder, that had been left over from the supply of the year before, was found, which, when administered to the sick, insured their recovery. This fact, at the time, gave additional perplexity to the problem, which in the end was so satisfactorily solved.

### *Friday, July 19, 1889.—Afternoon Session.*

The President, Dr. Henry A. Hull, in the chair.

The calling of the roll was dispensed with.

The report of the Board of Examiners was read.

The Board of Examiners respectfully submit the following:

We have carefully considered the recommendation of the National Association of Dental Examiners, and fully endorse their action in omitting the name of the *University of Maryland, Dental Department*, from the list of reputable colleges whose diplomas might be accepted instead of an examination, being fully convinced that they have not required a preliminary examination before matriculation, contrary to the rules required by this board and recommended by the National Association of Dental Examiners.

We have also decided to forward the evidence taken in the case to the National Association of Dental Examiners, and, before proceeding further, to await their action, as we understand from the dean of the Maryland University, Dental Department, that he hopes to have the college admitted to the National Association of Dental Faculties, and to comply with the rules and regulations governing that association and approved of by the National Association of Dental Examiners.

We also *fully* endorse the action of our representative at the Louisville meeting, August, 1888.

J. Hayhurst,

Fred. A. Levy,

James G. Palmer,

A. R. Eaton,

} *Board of Examiners.*

July 19, 1889.

Report adopted.

The Board of Examiners having reported favorably upon the application for membership of Dr. J. F. Lomas, of Bridgeton, a ballot was ordered, and Dr. Lomas was duly elected.

The Committee on President's Address reported as follows:

We heartily commend the excellent address of our president, and hope all will carefully read it when it shall have been published. But, as there is no special recommendation in it, we have no other report to make concerning it.

James G. Palmer,

C. S. Stockton,

Geo. E. Adams,

} *Committee.*

The amendment to the by-laws, making the dues of members three dollars a year, offered at the morning session, was taken up and adopted.

Dr. Luckey, chairman of the Committee on Dental Medicine, read a report, as follows:

So far as your committee has been able to learn, the past year has not been one of remarkable activity in therapeutic progress. Whilst nearly all dentists feel the need of a better understanding of the action of drugs, they have been devoting their time to the development of those already known rather than to experimenting with new ones. A good deal of time and thought has been given to cocaine, but to-day its position in the dental pharmacopœia is not materially altered from what it occupied a year ago. Iodoform is still a sheet-anchor with many, and its wonderful properties are often called into requisition for the benefit of sufferers and the gratification of operators. Iodol, the twin brother of iodoform, is being quietly but effectively used; while some have dropped both and have gone back to the old-time favorite in the treatment of pulpless and abscessed teeth,—iodide of creosote.

There seems to be a constant and not altogether unnatural tendency among practitioners for change. After a long series of successful cases with a medicine that has proved a faithful servant, an unsuccessful casé or a series of them persuades the operator that something else is needed, and forthwith a new remedy is called in.

The discovery of new remedies does not always imply progress, but the development of the properties of those already known to their highest and fullest possibilities, the discovery of their proper combination with other remedies, and the knowledge of the classes of cases upon which they may be used to as near a certainty as possible, does signify progress of a most satisfactory kind. The time was when many dentists thought that medicated cotton or other material was the proper thing for root-fillings, but progress has been indicated by the abandonment of such practice and the recognition of the fact that medicines are used to obtain a certain effect, and when that point has been reached it is time at once to discard the medicament and insert into the root some material destitute of active properties,—in other words, something inert. The point of cure has been reached. All that the tissues need is rest; and if allowed that rest, they will probably remain quiet and healthy; but the continued presence of any medicinal agent capable of correcting an abnormal state is likely to result eventually in a return of the very conditions which it was used to and did correct. Make a cure when possible, but then cease the use of medicines.

Listerine is growing more and more in favor. The experience of the chairman of your committee with it is very gratifying. As a wash in the treatment of pulpless teeth, during the process of removing the *débris* from the canal, it is unsurpassed; as a mouth-wash during the removal of tartar it is not only gratifying to the patient, but is also an excellent application for the gums. Its most useful and particular sphere, however, seems to be as a general mouth-wash in those cases where the teeth are soft, with white rings appearing at the cervical margins. These are the cases that above all others seem most discouraging to the operator, being apparently ready to soften on all surfaces and break down; where the only real remedy to stop decay is apparently the application of a gold crown to keep the oral fluids from the teeth and thus preserve them. Here listerine seems to be the very thing we need. Marked improvement has been observed in such cases after a six-months' course of treatment, and in a year all signs of softening have disappeared and the teeth appear strong and healthy. It is used in the strength of a teaspoonful to a half-glass of water, night and morning.

The rage for immediate root-filling still continues, and a word of warning in regard to this matter may not be out of place. If there are any cases that require our careful attention and the use of medicines, it is the treatment of pulpless or abscessed teeth. It may be justifiable to fill immediately the roots of a tooth the pulp of which has been recently destroyed or has died, or in cases where a fistulous opening exists; but in those cases where pus or other fluid exudates have no escape, the comfort of the patient and the successful issue of the case is jeopardized by immediate filling. Take a little time; insert your medicinal agent; give it a week at least to perform its work. If inflammation and swelling supervene,—not at all improbable in any case of long standing,—it is easy to reopen the tooth and search for the seat of the trouble. When everything is comfortable, fill the root, insert a temporary stopping in the cavity, and let the case rest another week, when, if the tooth is comfortable, it is perfectly safe to fill permanently. This method of procedure is much safer than the "rushing process," and may prevent the operator from having the mortification of undoing what to him seems a pretty piece of work. In this connection it is well to call attention to the fact that where inflammation appears in cases in which there is no fistulous opening, the use of the lance as a remedy is often very gratifying. Cut through the gum and process to the end of the root, including also the pericementum, and cut it from the apex fully one-half or more the length of the root. In many cases this is all that is required.

The care and treatment of pulpless teeth forms a large part of the practice of many dentists; and if a mistake is made in the treatment, no one can tell what disastrous consequences may follow. The immediate-root-filler may not hear of the subsequent result for a long time, but at last the word is likely to reach him that the tooth he has fondly imagined as successfully treated and saved has been extracted by some other dentist to afford relief to the afflicted patient.

A bad or unsuccessful result following immediate root-filling has a tendency to weaken the confidence of the public in such operations and operators; and many valuable teeth will be lost through dread from the failure of a few such hastily-performed operations. This warning is not uttered to impede progress, but to point out and emphasize some of the dangers that lie in the path of immediate-root-fillers.

The thing needed is a thorough understanding of the therapeutic value of the remedies we use, conjoined with the same understanding of the pathological and physiological condition of the tissues and parts we operate upon. Then, with a clear judgment to guide us, the outcome of any given case is not very doubtful.

*Dr. Watkins.*—Mr. President, Dr. Adair's paper is in the hands of the secretary. Its title is "Treatment of Very Sensitive Teeth when Pulps are nearly exposed."

On motion of Dr. Meeker, Dr. Adair's paper was read by title.

On motion of Dr. Meeker, Dr. Land's paper was read by title; Dr. Land not being present. Subject, "The Scientific Adaptation of Artificial Dentures."

On motion of Dr. Meeker, Dr. F. M. Odell's paper was read by title.

The officers-elect for the ensuing year were here formerly installed.

President Watkins takes the chair.

*Dr. Waters.*—Mr. President, I want to take this opportunity to thank the New Jersey State Dental Society for the very pleasant time, both intellectually and socially, which I have enjoyed at this meeting. I also desire to compliment this society upon the harmonious action I have observed in all its work. I have been particularly struck with your way of managing an election of officers. There was a good-fellowship and harmony through it all that I like to see. I have been in societies where there was a good deal of pulling and hauling on such occasions; I am glad to see that in the New Jersey State Dental Society that does not exist.

A vote of thanks was tendered to the gentlemen who read papers; to the visitors from a distance who came to take part in the discussion; to the exhibitors of dental appliances for their fine exhibits of things useful to the profession; and to the proprietors of the West End Hotel for courtesies extended.

Adjourned *sine die.*

### CLINICS.

*Dr. C. C. Carroll*, of New York, filled the root of a pulpless tooth with an aluminium pin, which subserves the purposes of perfectly stopping the root and making a retainer for the subsequent crown filling. In the next sitting the tooth filling will be finished with aluminium foil, which is claimed to be a superior tooth-saving material.

*Dr. W. H. Pomeroy*, of Gloucester, Mass., showed his method of condensing gold, which has grown out of experiments with the Herbst method; using the Pomeroy engine mallet, and soft gold, without annealing; the points used being smooth, and flattened on the end.

Dr. Pomeroy claims that his method of packing foil with flat points, combining the burnishing idea of Herbst with the mallet blow makes a filling more solid, and more like a gold ingot, than any other method, and that teeth can be filled as rapidly in that way as by the Herbst method.

*Dr. Wm. E. Truex*, of Freehold, filled with gold a crown cavity in a molar tooth.

*Dr. J. W. Canaday*, of Albany, N. Y., filled a left superior second molar, grinding surface, with a combination of tin and gold, a sheet of No. 4 gold and a sheet of No. 4 tin being folded together in such a way that the tin is completely enclosed by the gold. The combined material is used as soft gold would be, being burnished on as in the Herbst method.

*Dr. Reading*, of Lambertville, filled with crystal gold, and without separation, an approximal cavity in a central incisor; the operation occupying five minutes.

*Dr. J. Marion Edmunds*, of New York, filled a very large cavity in a left superior second molar, with hand pressure, using only the foil-carriers, and finishing with a plugger and burnisher; packing the gold so thoroughly that it could not be further condensed with the mallet. The gold used was crystalloid.

*Dr. C. A. Timme*, of New York, filled an approximal cavity in a superior second bicuspid with Wolrab's gold, in the mouth of Dr. Adelberg, using the Herbst burnishing method.

*Dr. Kimball* put in a cement filling, and demonstrated his method of lining cavities, where the pulp is nearly exposed.

*Dr. Edward J. King*, of Boonton, N. J., presented a very interesting case,—the removal of a fibrous tumor from a lad of seventeen, the operation having been performed by Dr. J. G. Ryerson. The tumor had distended the cheek very much, and after its removal a decided deformity presented, which was successfully remedied by Dr. King, by an ingenious piece of crown- and bridge-work. The description of the removal of tumor is not necessary here, and we regret we are unable to illustrate the case, as by that means alone could a correct idea be formed.

